# Glucagon-like Peptide Receptor Agonists and Kidney Outcomes in the Era of Personalized Medicine: Focus on Albuminuria

**DOI:** 10.3390/jpm16020097

**Published:** 2026-02-06

**Authors:** Ana Checa-Ros, Owahabanun Joshua Okojie, Jacob Gabriel Wassouf, Aida Yedean, Wei-Chung Hsueh, Patryk Hebda, Esther Rodriguez Llobell, Greta Bianca Muhmenthaler, Martin Duc-Duy Tran, Luis D’Marco

**Affiliations:** 1 Grupo de Investigación en Enfermedades Cardiorrenales y Metabólicas, Departamento de Medicina y Cirugía, Facultad de Ciencias de la Salud, Universidad Cardenal Herrera-CEU, CEU Universities, 46115 Valencia, Spain; ana.checaros@uchceu.es (A.C.-R.); joshua.okojie1@alumnos.uchceu.es (O.J.O.); 2 Aston Institute of Health & Neurodevelopment (AIHN), School of Life & Health Sciences, The Aston Triangle, Aston University, Birmingham B4 7ET, UK; 3 Snowball Project Group, Departamento de Medicina y Cirugía, Facultad de Ciencias de la Salud, Universidad Cardenal Herrera-CEU, CEU Universities, 46115 Valencia, Spain; jacob.wassouf@alumnos.uchceu.es (J.G.W.); aida.yedean1@alumnos.uchceu.es (A.Y.); weichung.hsueh@alumnos.uchceu.es (W.-C.H.); patryk.hebda@alumnos.uchceu.es (P.H.); esther.rodriguezllobell@alumnos.uchceu.es (E.R.L.); gretabianca.muhmenthaler@alumnos.uchceu.es (G.B.M.); martin.tran@alumnos.uchceu.es (M.D.-D.T.); 4 Department of Dentistry, Universidad Cardenal Herrera-CEU University, CEU Universities, 46115 Valencia, Spain

**Keywords:** GLP1-RA, albuminuria, diabetes, chronic kidney disease, precision medicine

## Abstract

The aim of this narrative review is to critically assess the renoprotective effects of glucagon-like peptide-1 receptor agonists (GLP-1RAs) in managing albuminuria among patients with type 2 diabetes mellitus within the framework of personalized medicine. By integrating current evidence from clinical trials and meta-analyses, the review highlights how GLP-1RAs not only enhance glycemic control but also reduce blood pressure, induce weight loss, and mitigate inflammatory responses. While these given factors may vary according to individual patient profiles, they also collectively contribute to slowing the progression of diabetic kidney disease (DKD). Additionally, the discussion emphasizes the dual cardiovascular and renal benefits from these agents, underscoring their role in reducing albuminuria and preserving renal function. The review also identifies gaps in knowledge, suggesting future research directions for optimizing patient selection and treatment regimens to maximize therapeutic benefits.

## 1. Introduction

Diabetic Nephropathy (DN) contributes greatly to the development of chronic kidney disease (CKD), being one of its leading causes [1]. In 2021, approximately 529 million people were affected by diabetes worldwide, and this is expected to rise to over 1.3 billion within the next thirty years [2]. According to the WHO, the mortality rate associated with diabetes has increased by 3% over the past two decades, resulting in approximately 284,049 deaths in 2019, predominantly impacting low- and middle-income countries [3].

Many factors are involved and play a substantial role in the development of DN, which include metabolic alterations, hemodynamic disorders, and dysregulation of hormone pathways, most especially angiotensin II, which itself, contributes to renal injury via oxidative stress [4]. Increase in oxidative stress due to long-term hyperglycemia is believed to augment proinflammatory protein levels, angiotensin II, protein kinase C activation, and transforming growth factor-beta (TGF-β) expression. This, in turn, stimulates local inflammation in the renal microenvironment, causing damage to nearby renal cells such as podocytes, mesangial cells, and endothelial cells, as well as causing local and systemic inflammation [5,6]. Thus, such injuries result in proteinuria and tubulointerstitial fibrosis. Increased angiotensin II levels further contribute to the production of reactive oxygen species/oxidative stress through activation of NADPH oxidase in the renal microenvironment [7]. Long-term hyperglycemia promotes renin and angiotensin II synthesis in mesangial cells, leading to elevated glomerular capillary pressure and permeability, which are key components in the development of proteinuria. This cascade also precipitates renal cell proliferation and hypertrophy, increased extracellular matrix synthesis, and subsequent macrophage activation and inflammation [5,7]. In addition to oxidative stress, local inflammation mediated by proinflammatory cytokines, such as interleukin–6 (IL-6), tumor necrosis factor-alpha (TNF-α), IL-18, can cause direct damage to the renal cells and can lead fibrosis via extracellular matrix accumulation. All these micro-damages with time would accumulate, thus leading to chronic and ultimately, end-stage kidney disease (ESKD).

Until recently, the main therapeutic options for the treatment of diabetes were insulin, biguanides, thiazolidinediones and sulfonylureas; with metformin (a biguanide) being the first-choice treatment [8]. But as of today, Glucagon-Like peptide 1 receptor agonists (GLP1-RAs), sodium-glucose cotransporter 2 inhibitors (SGLT2is), dipeptidyl peptidase 4 inhibitors (DPP4is) and mineralocorticoid receptor antagonists, are quickly becoming part of, if not the drugs of choice for diabetes, especially in cases with heart, kidney or liver comorbidities [9,10,11,12]. Specifically in the use of GLP-1RA, current clinical and genetic evidence indicate that individual characteristics such as body mass index (BMI), pharmacogenetic variants in the GLP-1 receptor and baseline cardiovascular risk profile can influence therapeutic response [13,14]. For example, patients with higher BMI may experience greater weight loss and metabolic improvements. While certain GLP-1R polymorphisms have been associated with glycemic and renal responses, underscoring the importance of individual characteristics in optimizing therapy [14,15,16]. Understanding these characteristics could help enable a precision medicine approach, allowing clinicians to tailor GLP-1RA therapy based on patient-specific factors. This individualized strategy can maximize reductions in albuminuria and slow CKD progression, while minimizing adverse effects [16,17].

The aim of this narrative review is to explore the current evidence on the renoprotective effects of GLP1-RAs, with a particular focus on their effects on albuminuria, and to discuss strategies for individualized implementation of these agents in the management of diabetic nephropathy.

## 2. Methodology

A comprehensive literature search was conducted using PubMed/MEDLINE, Scopus and Web of Science databases for articles published until November 2025. The search strategy combined keywords and Medical Subject Headings (MeSH) terms related to: (‘glucagon-like peptide 1 receptor agonist’ OR ‘GLP-1RA’ OR ‘incretins’) AND (‘diabetes mellitus’ OR ‘diabetes mellitus type 2’ OR ‘T2DM’ OR ‘diabetic kidney disease’) AND (‘albuminuria’ OR ‘non-albuminuric diabetic kidney disease’ OR ‘renal outcomes’ OR ‘renal pathology’) AND (‘precision medicine’ OR ‘personalized medicine’). Boolean operators (AND, OR) were used to refine the search. Original research articles (in vitro, in vivo, human observational and clinical trials) in which cardiovascular, renal and metabolic influences from GLP-1RA were the primary outcomes were included. Exclusion criteria included non-English language articles and studies not focusing on cardiovascular, renal or metabolic influences. The selection process was performed by all authors, focusing on several aspects of diabetic kidney disease and evaluating the overall benefits of GLP-1RAs. Evidence was critically appraised following the CASP checklist.

To improve transparency, we followed a PRISMA-inspired screening logic appropriate for a narrative/scoping review: records identified across databases were de-duplicated, screened by title/abstract, and assessed in full text for relevance to cardio-renal outcomes (albuminuria, eGFR trajectory) and the personalized medicine framework. Given the narrative/scoping scope, no protocol registration was performed, and we did not pool effect estimates; instead, we critically appraised key trials and meta-analyses and weighted interpretation by endpoint hierarchy (albuminuria as a surrogate vs. hard kidney outcomes).

## 3. Diabetic Kidney Disease

DN develops due to long-term hyperglycemia-induced damage at the microvascular level of the kidneys, which impairs the ability of the kidney to effectively excrete excess and waste products, eventually leading to reduced renal function and, in severe cases, ESKD [18]. Diabetic kidney disease (DKD), the clinical manifestation of DN, has an early phase called “incipient diabetic nephropathy”. During this initial stage, there is an increase in the permeability of the glomerular capillaries, which leads to loss of protein in the urine, especially albumin, commonly known as proteinuria or albuminuria with a corresponding parallel decrease in estimated glomerular filtration rate (eGFR) below 60 mL/min/1.73 m^2^ [19]. Whilst in non-albuminuric DKD, there is a decrease in the GFR below 60 mL/min/1.73 m^2^, but with an absence of albuminuria [19]. The decrease in kidney function with an absence of albuminuria, hints of damage to the tubule-interstitial parts of the kidney, whilst a decrease in kidney function with presence of albuminuria, hints of damage to the glomeruli parts of the kidney [20]. Albuminuria is one of the first clinical signs and serves as a crucial marker, indicative of kidney damage in patients with diabetes [21]. If not adequately managed, the condition progresses to DKD, marked by hypertension, exacerbated proteinuria, and a gradual decline in eGFR. In advanced stages, progressive renal fibrosis ensues, culminating in significant renal impairment and eventual ESKD [21]. In the early stages, DKD is typically asymptomatic [22]. As the disease progresses, clinical manifestations may emerge, including, but not limited to, refractory hypertension, peripheral or generalized edema, glaucoma, proteinuria characterized by foamy urine, and a range of systemic complications, such as cardiac, neurological and respiratory disorders [23,24,25,26]. Poor glycemic control, uncontrolled hypertension, and hypercholesterolemia have been identified as significant contributors to disease progression [27]. Additionally, diabetes vintage is a crucial determinant, with prolonged disease duration correlating with an increased risk of nephropathy onset [28]. A recent multicenter study demonstrated that early and aggressive intervention in DKD can delay the progression to ESKD, highlighting the importance of timely therapeutic measures [29]. Genetic susceptibility is a significant contributor to the development and progression of diabetic nephropathy, with certain individuals exhibiting increased risk due to inherited variants and familial clusters of renal phenotypes such as albuminuria and reduced glomerular filtration rate [30,31,32,33]. There genetic and phenotypic differences highlight the importance of personalized risk assessment and tailored therapeutic approaches in DKD [34,35]. Incorporating patient-specific factors, including genetic markers, metabolic profiles, and patterns of albuminuria, can facilitate earlier detection, improve prognostic accuracy and enable more precise selection of therapeutic interventions, moving clinical practice towards individualized management strategies [34,35,36,37].

## 4. Albuminuria as a Biomarker of Chronic Kidney Disease

**Figure 1:**
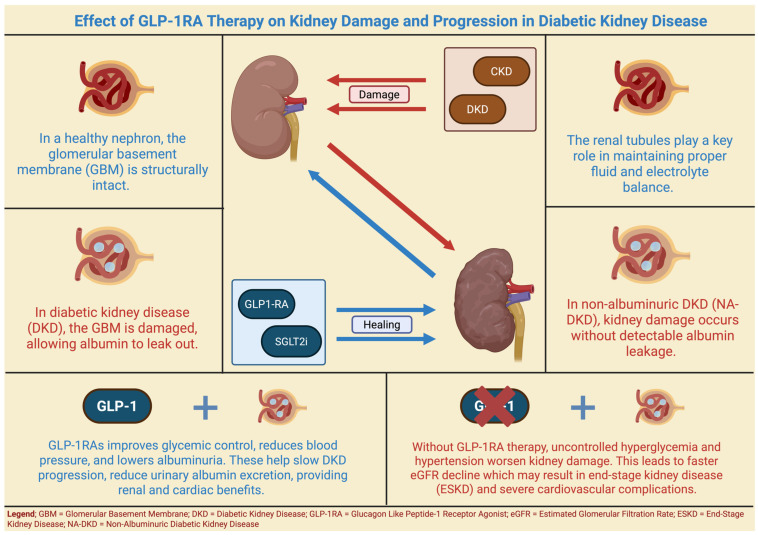
Effect of GLP-1RA Therapy on Kidney Damage and Progression in Diabetic Kidney Disease.

Albumin, a negatively charged globular protein synthesized by the liver, is essential for maintaining oncotic pressure. Albuminuria is defined as an abnormal excretion of albumin in urine [38]. It is determined via spot urinary albumin-to-creatinine ratio (UACR) and urine dipstick analysis, serving as a critical biomarker for the diagnosis, classification, and prediction of CKD and DKD. Uncovering the link between albuminuria, CKD and DKD can lead to a better understanding of the pathophysiology of the disease and provide insight into the timing of adequate treatment with new drugs, such as SGLT2i, GLP-1RAs and or mineralocorticoid receptor antagonist. Significant reductions in albuminuria are confirmed to correlate strongly with improved renal outcomes in diabetic patients, reinforcing its utility as a prognostic marker [39]. The glomerular capillaries of Bowman’s capsule are normally impermeable to proteins due to the electrostatic repulsion generated by negatively charged proteoglycans in the capillary walls (Figure 1). In CKD, damage to the renal vasculature, interstitium, and glomeruli alters the permeability and diminishes the negative charge of the glomerular basement membrane, thereby allowing proteins, especially albumin, to pass through the glomerular membrane (Figure 1) [40]. Studies have reported an incidence of albuminuria of approximately 77% in patients with type 1 diabetes mellitus (DM) with CKD and around 60% in those with type 2 DM [41]; in advanced CKD stages, the incidence reaches 75% [42]. Finally, there is a marked inverse relationship between albuminuria and CKD regression/clinical outcomes, meaning that the higher the levels of albuminuria, the lower the chances of the kidney regaining some of its function, and vice versa, thus making albuminuria a suitable biomarker of CKD [39]. Albuminuria further serves as a dynamic, individualized biomarker that could inform risk stratification and guide tailored management strategies. Baseline albuminuria levels, their trajectory over time, and the degree of response to interventions provide actionable data for optimizing therapy. Regular monitoring of albuminuria enables identification of patients most likely to benefit from specific reno-protective agents such as the SGLT2i, GLP-1RA or mineralocorticoid receptor antagonists mentioned above, and supports adjustment of treatment intensity to maximize benefit. The American Diabetes Association (ADA) and Kidney Disease: Improving Global Outcomes (KDIGO) recommend integrating albuminuria assessment into routine care to enhance early detection, refine therapeutic decisions and improve prediction of long-term renal and cardiovascular outcomes [43,44,45].

## 5. Non-Albuminuric Diabetic Kidney Disease Phenotype

DKD typically progresses through phases of glomerular hyperfiltration, microalbuminuria, overt proteinuria, and reduced eGFR, with albuminuria recognized as an early clinical marker [21,46]. However, a non-albuminuric DKD (NA-DKD) has been identified, accounting for approximately 20–40% of DKD cases, although studies reported that regional variability could exist [47]. It was described that the number of normoalbuminuric DKD patients has increased over the past two decades, as well as the prevalence of eGFR decline, meaning that the occurrence of DKD is stable and steadily on the rise [46]. NA-DKD may follow a distinct pathophysiological pathway, different from the classical phenotype, with NA-DKD having different macroscopic and microscopic characteristics and clinical outlooks, while the classical phenotype affects mainly the glomerulus, especially the podocytes [46,48]. Nevertheless, recent evidence has shown a more heterogeneous presentation of DKD, with many patients experiencing loss of kidney function without the presence of albuminuria, suggesting that both the onset and deterioration of renal function may also occur independently from the development of albuminuria [49]. The reason why the prevalence of the NA-DKD phenotype is on the rise is still unclear. It may be influenced by demographic changes, such as a surge in hypertension and obesity in an aging population, and advances in multifactorial treatment options, leading to improved glucose, lipid profile and blood pressure [47]. Additionally, the common use of renin–angiotensin-system (RAS) inhibitors in combination with SGLT2i and GLP1-RAs is playing a role in decreasing albuminuria by altering renal pathophysiological mechanisms [46,47]. The findings in albuminuric DKD are thickening of the glomerular basement membrane, mesangial matrix expansion, nodular lesions, glomerular sclerosis and arteriolar hyalinosis. In contrast, NA-DKD shows milder glomerular damage, as it tends to more severe tubulointerstitial and vascular lesions of varying arteriosclerotic degrees [19,46,47]. The identification of NA-DKD as a distinct clinical phenotype underscores the necessity for individualized patient evaluation and nuanced risk stratification. Reliance on albuminuria alone is insufficient for universal assessment of DKD progression; instead, a precision medicine approach should integrate additional factors such as the trajectory of eGFR, biomarkers of tubular injury, imaging findings and the presence of co-morbid conditions [50,51,52]. Understanding the mechanistic differences between albuminuric and non-albuminuric DKD enables more tailored therapeutic interventions. For example, GLP-1RAs may exert differential vascular and anti-inflammatory effects depending on DKD subtype, with emerging evidence suggesting potential benefits in NA-DKD [34,53,54]. Incorporating phenotype-specific insights into clinical practice helps facilitate the development of a more effective, patient-centered treatment strategy, moving beyond a one-size-fits-all paradigm [34,35].

## 6. GLP1-RAs: Pharmacological Characteristics

The discovery of GLP-1 in 1987 marked a significant advance in DM management [55]. GLP-1 is a gut hormone secreted by L-cells in the distal intestine in response to neural, nutritional, and hormonal stimuli, primarily postprandially. The process starts with a proglucagon molecule, which is finally cleaved into the active form, a 30 amino acid peptide, GLP-1 (7-36)-amide [56]. It is classified as an incretin hormone, exerting insulinotropic effects while suppressing glucagon secretion. Although the physiological effects of this naturally occurring molecule are short-lived due to its rapid degradation by the enzyme DPP-4, the wide variability of therapeutic benefits motivated the synthesis of GLP-1RAs. The most notable enhancements of the synthetic GLP-1RA include a longer half-life and improved stability [56]. It is important to note that the increase in insulin secretion occurs in a glucose-dependent manner, thus avoiding the incidence of hypoglycemic episodes. This is a distinguishing factor from the traditional, secretagogue class of drugs used in the treatment of Type 2 DM, which are not glucose-dependent and therefore carry a risk of hypoglycemia. Clinically, this makes GLP-1RA ideal for the management of Type 2 DM, which was the initial sole indication of this drug. Since then, its use has been expanded to other conditions that will be discussed below. GLP-1 receptors (GLP-1R) are G-protein coupled receptors predominantly located in pancreatic β cells, which underpins their role in glucose homeostasis. However, their expression in various tissues, albeit in smaller quantities, accounts for the multi-systemic effects of GLP-1RAs. One of the most notable effects is delayed gastric emptying, which contributes to decreased postprandial glucose peaks and increased satiety. While these effects are beneficial in metabolic regulation, they also underline the most reported adverse effects, namely, nausea, vomiting, and diarrhea, all gastro-intestinal related, particularly during the initiation or rapid dose escalation of therapy. Though these adverse effects may be common, they are not of severe or fatal nature. Additionally, within the central nervous system (CNS), GLP-1 administration has been associated with an anorectic response, although the precise mechanisms remain to be fully elucidated. These appetite-suppressing effects have contributed to the increasing use of GLP-1RAs in obesity management [57,58]. Beyond metabolic regulation, GLP-1RAs exhibit significant cardioprotective properties. GLP-1R are expressed in various cardiovascular cell types, including myocytes, endothelial cells, macrophages/monocytes, and smooth muscle cells [58]. Studies indicate that GLP-1R activation exerts anti-inflammatory effects on macrophages, reduces endothelial dysfunction, and inhibits smooth muscle proliferation. These mechanisms collectively attenuate atherosclerotic plaque formation, thereby reducing cardiovascular risk. Moreover, independent blood pressure reduction and plasma lipid modulation further contribute to cardiovascular protection [58,59]. Clinical evidence suggests that the use of GLP-1RAs is associated with a decrease in all-cause cardiovascular mortality [57,58,59].

More recently, emerging evidence has attributed nephroprotective properties to GLP-1RAs. While indirect benefits arise from improved glycemic control and weight loss, which alleviate renal hyperfiltration, direct renoprotective mechanisms have also been postulated. GLP-1R are expressed in both glomeruli and renal tubules, suggesting a direct effect on kidney function. The three primary mechanisms implicated in renal protection include modulation of renal hemodynamics, tubular function, and inflammation. Notably, GLP-1 receptor activation in afferent arterioles induces vasodilation, thereby enhancing glomerular filtration while simultaneously reducing intraglomerular pressure. This mechanism mitigates proteinuria by decreasing albumin leakage. At the tubular level, GLP-1 signaling inhibits profibrotic factor formation, thus reducing the progression of renal fibrosis. Furthermore, anti-inflammatory effects are achieved through two primary pathways—upregulation of the cyclic adenosine monophosphate–protein kinase A (cAMP–PKA) pathway and interference with advanced glycated end-products (AGEs). These processes collectively suppress reactive oxygen species (ROS) production, thereby attenuating the proinflammatory cascade that would otherwise lead to increased nuclear factor kappa-light-chain-enhancer of activated β-cells (NF-kB) expression and cytokine release. Overall, this evidence suggests that GLP-1RAs exert renoprotective effects that extend beyond glycemic control [60]. A randomized controlled trial by Mann J et al. [61] gives further support to the evidence that GLP-1RAs have beneficial effects on the kidneys. The study shows that in addition to improving glycemic control, GLP-1RAs reduce major adverse renal events in diabetic patients [61]. The clinical significance of these findings underscores the potential of GLP-1RAs in the prevention and management of DN.

Commonly prescribed GLP-1RA include Semaglutide (Ozempic^®^, Rybelsus^®^), Dulaglutide (Trulicity^®^), Liraglutide (Victoza^®^, Saxenda^®^), and Exenatide (Byetta^®^, Bydureon^®^) (Table 1). These agents differ in their half-life and route of administration, with both subcutaneous and oral formulations available, offering flexibility in therapeutic application. While generally well tolerated, gastrointestinal side effects remain the most frequently reported, particularly during initial dose escalation. These include diarrhea, nausea, and vomiting, especially when the initiation or titration occurs too rapidly. Despite these potential side effects, the overall benefits from GLP-1RAs in metabolic, cardiovascular, and renal health highlight their crucial role in the management of diabetes and its associated complications. Overall, GLP-1RAs possess diverse pharmacological properties that support individualized management of T2DM and related metabolic disorders. Their multifaceted mechanisms, including glucose-dependent insulin secretion, appetite regulation and cardio-renal protection, allow for therapeutic adaptation across patient subgroups, such as those with obesity, established cardiovascular disease or varying stages of DKD [62,63,64]. The availability of agents with distinct pharmacokinetic profiles, e.g., daily vs. weekly dosing and diverse receptor affinities and polymorphisms, enable clinicians to tailor regimens to optimize efficacy and minimize adverse effects [14,65,66].

## 7. GLP-1RA in Diabetes and Diabetic Kidney Disease

GLP-1RAs represent an advanced therapeutic option for type 2 DM, particularly in patients with concomitant cardiovascular and renal comorbidities. Hence, GLP-1RAs can be utilized as an adjunct alongside metformin as a first-line treatment for type 2 DM or, in specific cases, as a preferred therapeutic agent when additional benefits for cardiovascular or renal protection are required [84]. The therapeutic efficacy of GLP-1RAs is underscored by their ability to significantly lower glycated hemoglobin (HbA1c) levels, with reductions ranging from 0.42% to 1.6% depending on the formulation and duration of treatment. Although this reduction may appear modest, its long-term impact in preventing diabetes-related complications is substantial. Beyond glycemic control, GLP-1RA-induced weight loss, averaging 6.3 kg over 24–52 weeks, confers additional metabolic advantages, including improved lipid profiles, reduced insulin resistance, and decreased hypertension, each of which plays a crucial role in the prevention of diabetic complications [84]. The reduction in body weight achieved through GLP-1RA therapy holds significant implications beyond metabolic health. Obesity is a central component of metabolic syndrome and a major risk factor for hypertension, dyslipidemia, and insulin resistance. Consequently, weight loss facilitates improved blood pressure control and reduces the progression of albuminuria and DKD [85]. Furthermore, weight reduction has been associated with enhanced psychological well-being and adherence to treatment regimens, as patients who experience weight loss may be more motivated to adopt healthier lifestyles, including regular physical activity and improved dietary habits [86].

The antihypertensive effects of GLP-1RA have been well documented, with studies demonstrating significant reductions in both systolic and diastolic blood pressures [87]. While short-term administration of GLP-1RAs have been associated with a transient increase in basal heart rate, long-term therapy contributes to sustained reductions in blood pressure, which may enhance renal outcomes by mitigating hyperfiltration and endothelial dysfunctions. Although there have been reports of acute kidney injury in diabetic patients using GLP-1RAs, large-scale studies have not corroborated these concerns, and no substantial nephrotoxic effects have been identified [88]. Recent evidence suggests that GLP-1RAs may exert direct renoprotective effects independent of their metabolic benefits. These agents have been demonstrated to improve renal function by attenuating eGFR, reducing albuminuria, and decreasing oxidative stress. Studies in animal models have indicated protective effects against hypertension-induced kidney damage, while clinical trials, such as the LEADER trial, have confirmed the renal and cardiovascular benefits of GLP-1RA, particularly in patients with CKD [89] (Figure 1). Additionally, GLP-1RAs may activate receptors in cardiac myocytes, leading to the secretion of atrial natriuretic peptide (ANP), which promotes natriuresis, vasodilation, and inhibition of the RAAS [90]. This mechanism further supports the role of GLP-1RAs in blood pressure regulation and nephroprotection [87]. A meta-analysis reinforces the renoprotective effects of GLP-1RAs, demonstrating significant reductions in composite renal outcomes among patients with type 2 DM [91]. The multifaceted benefits from GLP-1RAs extend beyond glycemic control to include substantial improvements in cardiovascular and renal health, positioning these agents as a cornerstone of modern diabetes management. In addition, current consensus in the medical literature positions GLP-1RAs as a cornerstone of modern diabetes management, especially for patients with high cardiovascular or renal risk [92,93]. Their integration into precision medicine approaches allows for individualized treatment strategies based on patient-specific characteristics, optimizing outcomes in metabolic, cardiovascular and renal domains [93,94].

## 8. GLP-1RA and Renal Outcomes

The standard therapy for DKD primarily focuses on optimal glycemic and blood pressure control, aimed at preventing disease progression and reducing albuminuria, a strong predictor of eGFR decline [7,95]. Other therapeutic strategies for managing DKD include weight loss, protein restriction, lipid control, and smoking cessation. The evidence has highlighted the renoprotective effects of GLP-1RAs, such as Liraglutide, Dulaglutide, and Semaglutide, in patients with type 2 DM at risk for CVD, particularly in terms of reducing albuminuria and improving glycemic control [96,97]. These therapeutic agents are believed to have a significant effect in reducing adverse renal outcomes by targeting various hemodynamic and metabolic factors that contribute to disease progression [96,98]. Liraglutide, a GLP-1RA, has been shown to significantly reduce HbA1c, body weight, and systolic blood pressure (SBP) in patients with Type 2 DM and CKD, leading to a reduction in albuminuria [99]. Early-stage studies revealed an initial increase in 24 h ambulatory SBP, which subsided over time, leading to a net reduction in SBP. Additionally, treatment with Liraglutide was associated with reduced mean baseline urinary sodium, extracellular volume, natriuresis, N-terminal pro b-type natriuretic peptide (NT-proBNP) and urinary albumin excretion rate, all of which are indicative of its beneficial effects on renal function [87]. Furthermore, the reduction in urinary albumin excretion and the potential for micro- and macroalbuminuria regression support the renoprotective effects of GLP-1RAs, attributed to their anti-glycemic and antihypertensive properties. GLP-1RAs also exhibit inhibitory effects on Angiotensin II (Ang-II) and its proinflammatory actions in the glomerulus, providing an additional layer of protection against kidney damage [100].

Landmark cardiovascular outcome trials (CVOTs) provided the first consistent signals of renal benefit with GLP-1RAs, largely driven by exploratory composite kidney endpoints and reductions in new or worsening albuminuria (e.g., LEADER, REWIND, SUSTAIN-6, EXSCEL, and ELIXA; and AMPLITUDE-O for efpeglenatide) [91,101,102,103,104,105]. These trials were not primarily powered for hard kidney outcomes (eGFR slope, kidney failure, kidney replacement therapy), but they established albuminuria lowering as a reproducible effect and framed subsequent kidney-focused development programs. Most importantly, the FLOW trial represents a major milestone by testing semaglutide in patients with chronic kidney disease and type 2 diabetes, moving beyond exploratory renal signals toward kidney-specific endpoints [29].

In the LEADER trial, Liraglutide demonstrated substantial cardiovascular and renal benefits in patients with Type 2 DM and CKD, while maintaining a favorable safety profile [91]. The trial showed a reduced risk of severe hypoglycemia, bone and joint injuries, and acute kidney injury compared to placebo, further confirming the safety and efficacy of Liraglutide in this patient population [91]. Notably, despite the positive effects on renal outcomes, the study also reported an increased incidence of gastrointestinal side effects, including nausea, vomiting, and diarrhea, though these were not statistically significant [89].

Beyond traditional GLP-1RAs, dual and emerging multi-agonists may have additional metabolic and renal implications. Tirzepatide combines GLP-1 and glucose-dependent insulinotropic polypeptide (GIP) receptor agonism [106]; in a post hoc analysis of SURPASS-4 (tirzepatide vs. insulin glargine in high cardiovascular-risk type 2 diabetes), tirzepatide was associated with a reduction in albuminuria whereas insulin glargine showed an increase, supporting the hypothesis that greater weight loss and metabolic improvements can translate into larger albuminuria reductions [107]. Triple agonists (e.g., retatrutide: GLP-1/GIP/glucagon receptor agonism) are under active investigation and may further amplify cardiometabolic benefits [108]; however, kidney outcomes remain preliminary. Ongoing trials such as REMODEL should help clarify longer-term renal signals and the extent to which albuminuria changes translate into durable structural kidney protection [109].

Another key trial, REWIND, evaluated the long-term effects from Dulaglutide in patients with Type 2 DM. The results demonstrated a significant reduction in composite renal outcomes, largely driven by the improvements in glycemic control and reductions in SBP, which in turn led to a decreased risk of eGFR decline and cardiovascular events [110]. These findings further support the reno and cardioprotective benefits from GLP-1RAs. Overall, the combined anti-glycemic and antihypertensive actions of GLP-1RAs are key factors contributing to their beneficial effects on renal outcomes in patients with Type 2 DM. These treatments significantly reduce urinary albumin excretion, lower SBP, and improve HbA1c levels. Additionally, the effects on renal function, such as changes in eGFR, appear to be reversible upon discontinuation, suggesting a hemodynamic rather than structural mechanism. Long-term follow-up data from trials have confirmed the sustained renal benefits of GLP-1RA therapy, demonstrating a significant reduction in the rate of eGFR decline among treated patients [110]. Finally, GLP-1RA are emerging as a critical therapeutic option in the prevention and management of DKD. Their ability to improve glycemic control, reduce blood pressure, and modulate renal hemodynamics provides substantial reno- and cardioprotective effects, making them a valuable addition to the therapeutic armamentarium for managing Type 2 DM and associated renal complications.

Beyond its proven benefits in managing obesity and reducing cardiovascular risk, as demonstrated in SELECT Trial [111], Semaglutide also represents an important therapeutic option for CKD in patients with Type 2 DM [29]. In the FLOW trial, which enrolled 3533 patients, treatment with Semaglutide (Table 2), a 24% reduction was observed in the urine albumin-to-creatinine ratio, alongside a decreased incidence of acute kidney injury, attenuation of CKD progression and a lower risk of progression to kidney transplantation. Also, Semaglutide was shown to have lower risk of major adverse cardiovascular outcomes, like heart failure, in comparison with the placebo [29]. Considering that most of the deaths in such patients result from cardiovascular complications, Semaglutide may prolong CKD patients’ lives. Perkovic et al. [29] mentioned that this could be due to possible anti-inflammatory routes activated by GLP-1RAs. Given that GLP-1RAs decrease production and release of pro-inflammatory cytokines, many kidney cells and immune cells which have GLP-1 receptors would thus be suppressed from promoting inflammation and so, this could be one of the reasons for the reduction in albuminuria [29,112]. In essence, GLP-1RAs have a strong beneficial effect on albuminuria, especially in relation to T2DM (Table 2).

The randomized controlled trials (RCTs) summarized in Table 2 consistently demonstrate that GLP-1RAs, such as exenatide, semaglutide, and dulaglutide, significantly reduce albuminuria in patients with T2DM and CKD. Across studies, treatment with GLP-1RAs led to reductions in urinary albumin excretion rate (UAER) or urine albumin-to-creatinine ratio (UACR), with improvements ranging from 24% to 29.7% in reductions. These beneficial effects were observed over follow-up periods ranging from 6 months to 3.4 years, indicating both short- and long-term potential for GLP-1RAs in managing albuminuria in diabetic nephropathy. While the meta-analyses also show positive effects on managing albuminuria in similar patients. Across various studies, GLP-1RAs led to a significant decrease in urinary albumin excretion, with relative risks ranging from 0.82 to 0.85, indicating a reduced progression of albuminuria. The duration of follow-up varied from 5 weeks to over 3 years, once again demonstrating both short-term and long-term benefits of GLP-1RAs in managing albuminuria and improving kidney outcomes.

Table 3 compares the four major renoprotective drug classes, namely GLP-1RAs, SGLT2 inhibitors, Aldosterone antagonists and RAAS inhibition (ACEi/ARB), all in the context of treating albuminuria due to diabetic kidney disease. The table shows that SGLT2is and aldosterone antagonists provide the most robust and additive reductions in albuminuria and CKD progression, with SGLT2is showing consistent benefit across all albuminuria and eGFR levels [122]. RAAS inhibitors remain foundational, especially for albuminuric CKD, while GLP-1RAs offer modest albuminuria reduction and strong cardiovascular benefit, but less direct renal protection compared to SGLT2is [123]. Combination therapy (SGLT2i plus aldosterone antagonist) yields greater albuminuria reduction than either agent alone [124].

## 9. GLP-1RA and Precision Medicine

Precision medicine strategies are evolving to incorporate genetic, metabolic, and clinical biomarkers to optimize GLP-1 RA therapy, aiming to maximize efficacy and minimize adverse effects. Ongoing research is focused on developing predictive models and identifying subgroups most likely to benefit, including those with specific genetic variants, comorbidities, or risk profiles [65,66,129]. The integration of these insights into clinical practice is expected to improve long-term outcomes and patient satisfaction in diabetes and obesity management. Metabolic biomarkers, including measures of β-cell function, insulin sensitivity, BMI, and liver fat, are being integrated into predictive models to further refine patient selection. These models, often leveraging machine learning, combine genomic, proteomic, and clinical data to stratify patients by likelihood of response, with recent studies achieving high accuracy in distinguishing responders from non-responders [130,131,132]. Structural pharmacology advances are enabling the design of biased agonists and allosteric modulators, which may selectively engage beneficial signaling pathways while reducing side effects [133,134].

Importantly, in patients receiving GLP-1/GIP (and emerging multi-agonist) therapies with substantial weight loss, creatinine-based eGFR equations may overestimate kidney function due to reductions in muscle mass and creatinine generation. Where available, cystatin C-based eGFR (or combined creatinine-cystatin C equations) should be considered to improve accuracy, particularly in longitudinal follow-up and trial interpretation.

The latest near-clinical-use genetic and metabolic biomarkers for predicting individual response to GLP-1RA therapy include the following;

1. GLP-1R gene polymorphisms: Variants in the GLP1R gene, such as rs6923761 and rs10305420, have been associated with differential glycemic and weight responses to GLP-1RA therapy. Carriers of certain alleles may experience greater reductions in HbA1C and body weight, while others may have attenuated responses or increases risk of GI side effects [14,66,133,135,136].
2. Other genetic loci: Polymorphisms in genes such as CNR1, CTRB1/2 andTCF7L2, have shown associations with GLP-1RA response, particularly liraglutide, affecting glycemic control and metabolic parameters [14,133,135].
3. Proteomic and metabolomic markers: Machine learning approaches have identified panels of proteomic markers and metabolomic profiles that predict GLP-1RA response with high accuracy. These omics-based biomarkers are promising for future clinical stratification, but require validation and standardization before widespread adoption [130,137].
4. Clinical phenotype-based algorithms: Recent work using large real-world datasets and Bayesian causal forest modeling has enabled individualized prediction of glycemic response to GLP-1RA vs. SGLT2i based on routine clinical features (e.g., sex, BMI, baseline HbA1c). Notably, women show a greater glycemic response to GLP-1RA, and phenotype-based algorithms are being piloted for treatment selection in clinical practice [131,138].

But, despite these advances, clinical implementation of precision GLP-1RA therapy is limited by the lack of validated predictive biomarkers and standardized patient classification systems [139]. Current research is focused on validating these predictive models in diverse populations and expanding biomarker panels to include polygenic risk scores and proteomic signatures. The goal is to enable personalized GLP-1RA therapy that maximizes efficacy, minimizes adverse effects, and improves long-term outcomes in type 2 diabetes and obesity management [129,140].

In terms of real-world implementations, currently, the standard of care 1st line treatment for renal affections in diabetic patients is SGLT2i, if the eGFR is adequate. GLP-1RAs are recommended when SGLT2i are contraindicated, insufficient or not well tolerated [141]. Some studies confirm that initiation of GLP-1 receptor agonists is associated with a lower risk of albuminuria progression and a less steep decline in eGFR compared to basal insulin or DPP-4 inhibitors, with hazard ratios for new macroalbuminuria events ranging from 0.80 to 0.89 [142]. However, when compared to SGLT2 inhibitors, GLP-1 receptor agonists are less effective for kidney outcomes, including albuminuria and eGFR decline, but remain superior to DPP-4 inhibitors, sulfonylureas, and basal insulin [143,144]. Thus, currently, SGLT2i remains the 1st choice of treatment.

GLP-1RAs are also substantially more expensive than older agents, and their subcutaneous administration may limit uptake in some populations. Nonetheless, their favorable safety profile in CKD (no dose adjustment required for most agents) and the absence of increased hypoglycemia risk support their use in appropriate patients [115,145]. Gastrointestinal side effects are the most common dose-limiting adverse events, and careful titration is recommended to optimize tolerability [145].

## 10. Conclusions

In summary, GLP-1RAs offer a promising therapeutic strategy for type 2 diabetes patients, especially those with or at risk for early CKD. They provide integrated benefits including improved glycemic control, reduced albuminuria, lowering blood pressure, and weight loss. Nonetheless, their role in preventing ESKD and impacting non-albuminuric DKD shows potential; long-term data on structural renal benefits are still needed. Personalized medicine guided by albuminuria, eGFR, and individual patient factors is essential. Finally, a multidisciplinary approach and future research focusing on long-term outcomes and optimal patient selection will help maximize the renal and cardiometabolic benefits of GLP-1RAs.

We emphasize that the most consistent renal signal across the available evidence is albuminuria reduction; although albuminuria is a clinically meaningful biomarker and treatment target, it remains a surrogate endpoint and does not, by itself, prove reduction in kidney failure or need for kidney replacement therapy. Accordingly, claims of long-term structural renoprotection should be interpreted cautiously until kidney-focused trials and longer follow-up data confirm benefits on hard renal endpoints (eGFR slope, ESKD). Precision medicine approaches (genetics, omics, and machine-learning models) are promising but remain largely exploratory and require external validation and standardization prior to routine clinical implementation.

## Figures and Tables

**Table 1:** Comparison of GLP-1 Receptor Agonists, their pharmacological and clinical characteristics.

Drug	Molecule	Manufacturer/Brand Name	FDA Approval Date	Treatment Indications	Route of Administration	Mechanism of Action	Half-Life/Dosage Regimen	Adverse Effects	Contraindications
Semaglutide	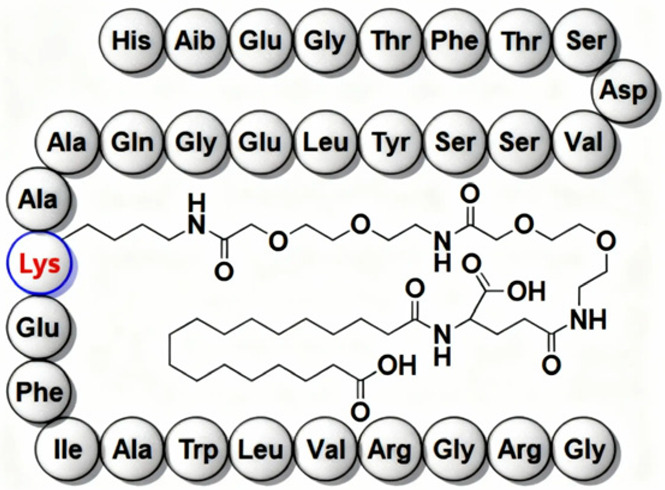 [67]	Novo Nordisk/Ozempic [68], Wegovy [69], Rybelsus [70]	Ozempic: 2017 [68]; Wegovy: 2024 [69]; Rybelsus: 2017 [70]	Type 2 Diabetes Mellitus: Ozempic, Rybelsus [68,70]; Obesity: Wegovy [69]	Subcutaneous: Ozempic, Wegovy [68,69]; Oral: Rybelsus [70]	GLP-1 receptor agonist [68,69,70]	165–184 h/once weekly [68,69]; 24 h/once daily [70]	GI symptoms (nausea, vomiting, indigestion, diarrhea), pancreatitis, alopecia [68,69,70]	Medullary thyroid cancer risk based on rodent data, Personal or family history of medullary thyroid carcinoma or in patients with Multiple Endocrine Neoplasia syndrome type 2 (MEN2) [68,69,70]
Exenatide	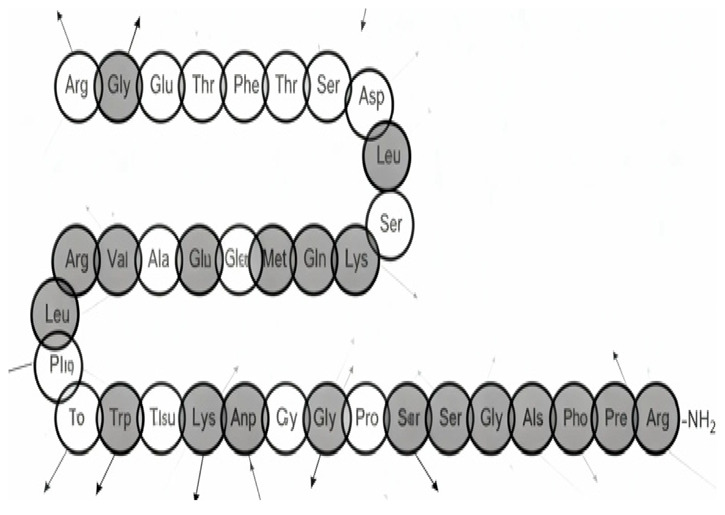 [71]	AstraZeneca/Byetta [72], Bydureon [73]	Byetta: 2005 [72]; Bydureon: 2005 [73]	Type 2 diabetes [72,73]	Subcutaneous [72,73]	GLP-1 receptor agonist [72,73]	2.4 h/twice daily [72,73]	GI symptoms (nausea, vomiting, indigestion diarrhea), pancreatitis [72,73]	Medullary thyroid cancer risk based on rodent data, Personal or family history of medullary thyroid carcinoma or in patients with Multiple Endocrine Neoplasia syndrome type 2 (MEN2) [72,73]
Liraglutide	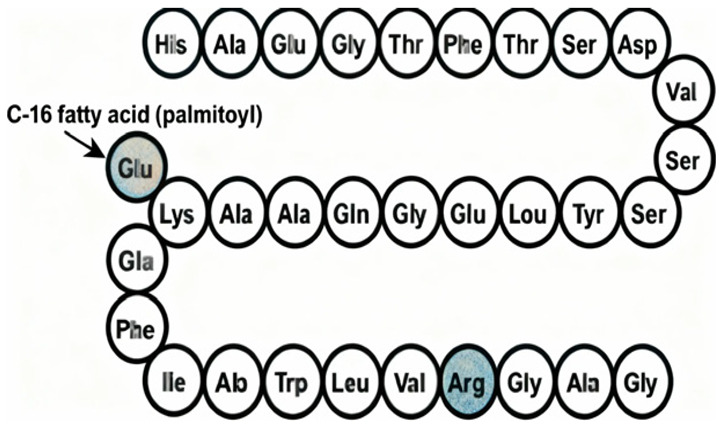 [74]	Novo Nordisk/Victoza [75], Saxenda [76]	Victoza: 2010 [75]; Saxenda: 2010 [76]	Type 2 diabetes [75,76]	Subcutaneous [75,76]	GLP-1 receptor agonist [75,76]	13 h/once daily [75,76]	GI symptoms (nausea, vomiting, indigestion, diarrhea) [75,76]	Medullary thyroid cancer risk based on rodent data, Personal or family history of medullary thyroid carcinoma or in patients with Multiple Endocrine Neoplasia syndrome type 2 (MEN2) [75,76]
Dulaglutide	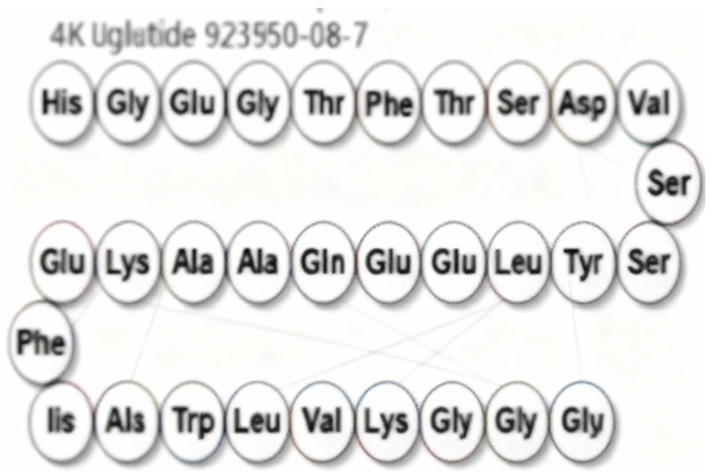 [77]	Eli Lilly/Trulicity [78]	2014 [78]	Type 2 diabetes [78]	Subcutaneous [78]	GLP-1 receptor agonist [78]	120 h/once weekly [78]	GI symptoms (nausea, vomiting, indigestion, diarrhea) [78]	Medullary thyroid cancer risk based on rodent data, Personal or family history of medullary thyroid carcinoma or in patients with Multiple Endocrine Neoplasia syndrome type 2 (MEN2) [78]
Lixisenatide	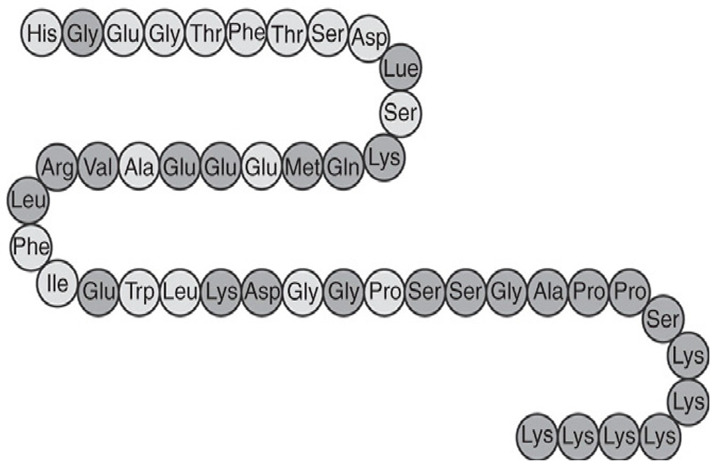 [79]	Sanofi/Lyxumi/Adlyxin [80]	2016 [80]	Type 2 diabetes [80]	Subcutaneous [80]	GLP-1 receptor agonist [80]	2–4 h, once daily [80]	GI symptoms (nausea, vomiting, indigestion, diarrhea) [80]	Medullary thyroid cancer risk based on rodent data, Personal or family history of medullary thyroid carcinoma or in patients with Multiple Endocrine Neoplasia syndrome type 2 (MEN2) [80]
Tirzepatide	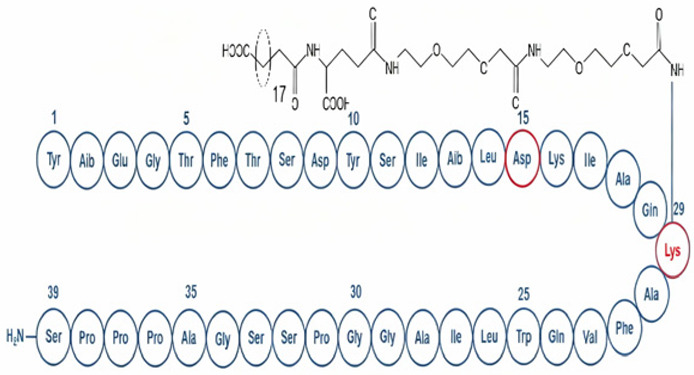 [81]	Eli Lilly/Mounjaro [82], Zepbound [83]	Mounjaro: 2022 [82]; Zepbound: 2024 [83]	Type 2 diabetes: Mounjaro [82]; Obesity: Zepbound [83]; Obstructive sleep apnea: Zepbound [83]	Subcutaneous [82,83]	GLP-1 receptor agonist + Gastric inhibitory polypeptide [82,83]	120 h, once weekly [82,83]	GI symptoms (nausea, vomiting, indigestion, diarrhea) [82,83]	Medullary thyroid cancer risk based on rodent data, Personal or family history of medullary thyroid carcinoma or in patients with Multiple Endocrine Neoplasia syndrome type 2 (MEN2) [82,83]

Legend; GLP-1 = Glucagon-Like Peptide-1; GI = Gastro-Intestinal.

**Table 2:** Summary of Randomized and Meta-analyses on GLP-1 Receptor Agonists and albuminuria.

Study	Study Type	CASP Score	Quality	GLP-1 Receptor Agonist	Baseline Population	Baseline Urine Albumin-Creatinine Ratio or Urinary Albumin Excretion Rate	Controls	Method of Albuminuria Assessment	Change in Albuminuria	Follow-Up Period
1, Wang et al., 2020 [113]	RCT	7	Moderate	Exenatide	T2DM patients with eGFR> or equal to 30 and macroalbuminuria	1512 mg/24 h	Insulin lispro plus glargine	Using percentage change in the urinary albumin excretion rate (UAER)	29.7% reduction in urinary albumin excretion rate (*p* = 0.0255)	24 weeks
2, Perkovic et al., 2024 [29]	RCT	10	High	Semaglutide	T2DM patients with chronic kidney disease (defined by an estimated glomerular filtration rate [eGFR] of 50 to 75 mL per minute per 1.73 m^2^ of body-surface area and a urinary albumin-to-creatinine ratio [with albumin measured in milligrams and creatinine measured in grams] of >300 and <5000 or an eGFR of 25 to <50 mL per minute per 1.73 m^2^ and a urinary albumin-to-creatinine ratio of >100 and <5000)	567.6 mg/g	Placebo	Using percentage change in the urinary albumin excretion rate (UAER)	24% reduction in urine albumin-to-creatinine ratio over 2 years (pooled analysis); 24% reduction in primary composite outcome (hazard ratio 0.76, 95% confidence interval 0.66–0.88)	3.4 years
3, Shaman et al., 2022 [114]	RCT	7	Moderate	Semaglutide, Liraglutide	T2DM patients at high risk of cardiovascular events and with a glycohemoglobin (HbA1c) level ≥ 7%.	No mention found	Placebo	Using percentage change in the urinary albumin excretion rate (UAER)	24% reduction in albuminuria at 2 years (95% confidence interval 20–27%)	2–3.8 years
4, Natale et al., 2025 [115]	Meta-analysis	10	High	Multiple agonists were used	T2DM patients with CKD randomly allocated to a GLP-1 receptor agonist, placebo, standard care or a second glucose-lowering agent. CKD included all stages (from 1 to 5)	No mention found	Placebo	Using percentage change in the urinary albumin excretion rate (UAER)	Reduced albuminuria progression (relative risk 0.85, 95% confidence interval 0.74–0.98)	26 weeks
5, Aart-van der Beck et al., 2020 [116]	RCT	8	Moderate	Exenatide	T2DM patients with CKD and baseline uACR ≥ 30 mg/g	≥30 mg/g	Oral glucose-lowering drugs (including insulin [insulin glargine, insulin detemir] or oral antidiabetic drugs [OADs; sitagliptin, metformin or pioglitazone] in patients with type 2 diabetes)	Using percentage change in the urinary albumin excretion rate (UAER)	26.2% reduction in urine albumin-to-creatinine ratio (95% confidence interval −39.5 to −10)	26–28 weeks
6, Badve et al., 2025 [117]	Meta-analysis	10	High	Multiple agonists were used	Patients with T2DM	No mention found	Placebo	Using percentage change in the urinary albumin excretion rate (UAER)	18% reduction in composite kidney outcome (hazard ratio 0.82, 95% confidence interval 0.73–0.93)	≥12 months
7, Konstantina et al., 2022 [118]	RCT	7	Moderate	Multiple agonists were used	T2DM patients with chronic kidney disease	No mention found	Placebo	Using percentage change in the urinary albumin excretion rate (UAER)	25% reduction in macroalbuminuria (95% confidence interval 19–32%)	≥6 months
8, Mendonça et al., 2025 [119]	Meta-analysis	9	High	Multiple agonists were used	Patients with type 2 diabetes or with overweight/obesity status, with or without CKD, with kidney events reported as primary or secondary end points.	No mention found	Placebo	Using percentage change in the urinary albumin excretion rate (UAER)	Reduced risk of worsening kidney function (relative risk 0.84, 95% confidence interval 0.77–0.91)	No mention found
9, Tuttle et al., 2017 [120]	RCT	8	Moderate	Dulaglutide	4.4% (*n* = 265) of participants had persistent eGFR < 60 mL/min/1.73 m^2^, 3% (*n* = 181) had persistent macroalbuminuria (defined as UACR > 300 mg/g), and 7.1% (*n* = 425) had eGFR < 60 mL/min/1.73 m^2^ and/or macroalbuminuria.	No mention found	Placebo, exenatide, insulin glargine, metformin and sitagliptin	Using percentage change in the urinary albumin excretion rate (UAER)	Lower urine albumin-to-creatinine ratio versus placebo/active comparators (*p* = 0.023–0.029)	26–52 weeks
10, Li et al., 2025 [121]	Meta-analysis	9	High	Multiple agonists were used	T2DM patients with any condition or disease	No mention found	Placebo or other hypoglycemic agents	Using percentage change in the urinary albumin excretion rate (UAER)	Decreased urine albumin-to-creatinine ratio (weighted mean difference −0.10, 95% confidence interval −0.19 to −0.01)	5 weeks–3.84 years

Legend; GLP-1 = Glucagon-Like Peptide-1; T2DM = Type 2 Diabetes Mellitus; UAER = Urinary Albumin Excretion Rate.

**Table 3:** A comparison of GLP-1RAs and other renoprotective therapies.

Drug Class	Effect on Albuminuria	Effect on eGFR/CKD Progression	Effect on ESKD	Cardiovascular Outcomes	Safety/Adverse Events
GLP-1 Receptor Agonists (GLP-1RAs)	Reduces albuminuria [29,61,110]	Slows eGFR decline [29,89,110]	No clear reduction in ESKD; driven by albuminuria reduction [29,61]	Reduces MACE, all-cause mortality [57,91,96]	GI side effects (nausea, vomiting)
SGLT2 Inhibitors (SGLT2i)	Robust reduction (30–50%); effect across albuminuria levels [122]	Slows eGFR decline; benefit across CKD stages and albuminuria [122,125]	Reduces ESKD risk [125]	Reduces MACE, HF hospitalization [122]	Genital mycotic infections, rare DKA
Aldosterone antagonist (Finerenone)	Significant reduction (30–50%) [126]; additive with SGLT2i [125]	Slows eGFR decline; reduces CKD progression [126,127]	Reduces ESKD risk [126]	Reduces CV events [127]	Hyperkalemia (especially with low eGFR)
RAAS Inhibition (ACEi/ARB)	Reduces albuminuria; prevents progression from micro- to macroalbuminuria [43,128]	Slows CKD progression [43,128]	No proven benefit for ESKD in low albuminuria [128]	Reduces CV events [43]	Hyperkalemia, increased creatinine

Legend; eGFR = estimated Glomerular Filtration Rate; CKD = Chronic Kidney Disease; ESKD = End-Stage Kidney Disease; MACE = Major Adverse Cardiac Events; HF = Heart Failure; CV = Cardiovascular; DKA = Diabetic Ketoacidosis.

## Data Availability

No new data were created or analyzed in this study. Data sharing is not applicable to this article.

## Abbreviations

The following abbreviations are used in this manuscript:

AGEs	Advanced Glycated End-products
Ang-II	Angiotensin II
ANP	Atrial natriuretic peptide
CNS	Central Nervous System
CKD	Chronic Kidney Disease
CVD	Cardio-Vascular Disease
cAMP-PKA	Cyclic Adenosine Monophosphate–Protein Kinase A
DKD	Diabetic Kidney Disease
DM	Diabetes Mellitus
DN	Diabetic Nephropathy
DPP4i	Dipeptidyl Peptidase 4 Inhibitor
ESKD	End-Stage Kidney Disease
eGFR	Estimated Glomerular Filtration Rate
GI	Gastro-Intestinal
GLP-1	Glucagon-Like Peptide 1
GLP-1R	Glucagon-Like Peptide 1 Receptor
GLP1-RA	Glucagon-Like Peptide 1 Receptors Agonist
HbA1c	Hemoglobin A1C
IL-6	Interleukin-6
IL-18	Interleukin-18
NA-DKD	Non-Albuminuric DKD
NT-proBNP	N-terminal pro B-type Natriuretic Peptide
NF-kB	Nuclear Factor kappa-light-chain-enhancer of activated B-cells
RAAS	Renin–Angiotensin–Aldosterone System
ROS	Reactive Oxygen Species
SGLT2i	Sodium-Glucose co-transporter 2 Inhibitor
SBP	Systolic Blood Pressure
TGF-beta	Transforming Growth Factor-beta
TNF-alpha	Tumor Necrosis Factor-alpha
T2DM	Type 2 Diabetes Mellitus
UACR	Urinary Albumin-to-Creatinine Ratio
WHO	World Health Organization
